# Adiponectin signalling in bone homeostasis, with age and in disease

**DOI:** 10.1038/s41413-020-00122-0

**Published:** 2021-01-07

**Authors:** Jonathan W. Lewis, James R. Edwards, Amy J. Naylor, Helen M. McGettrick

**Affiliations:** 1 Rheumatology Research Group, Institute of Inflammation and Ageing, University of Birmingham, Birmingham, B15 2TT UK; 2 Ageing & Regeneration Research Group, Botnar Research Centre, University of Oxford, Oxford, OX3 7LD UK

**Keywords:** Bone, Pathogenesis, Endocrinology

## Abstract

Adiponectin is the most abundant circulating adipokine and is primarily involved in glucose metabolism and insulin resistance. Within the bone, osteoblasts and osteoclasts express the adiponectin receptors, however, there are conflicting reports on the effects of adiponectin on bone formation and turnover. Many studies have shown a pro-osteogenic role for adiponectin in in vivo murine models and in vitro: with increased osteoblast differentiation and activity, alongside lower levels of osteoclastogenesis. However, human studies often demonstrate an inverse relationship between adiponectin concentration and bone activity. Moreover, the presence of multiple isoforms of adiponectin and multiple receptor subtypes has the potential to lead to more complex signalling and functional consequences. As such, we still do not fully understand the importance of the adiponectin signalling pathway in regulating bone homeostasis and repair in health, with age and in disease. In this review, we explore our current understanding of adiponectin bioactivity in the bone; the significance of its different isoforms; and how adiponectin biology is altered in disease. Ultimately, furthering our understanding of adiponectin regulation of bone biology is key to developing pharmacological and non-pharmacological (lifestyle) interventions that target adiponectin signalling to boost bone growth and repair in healthy ageing, following injury or in disease.

The principal cellular constituents of bone (primarily osteoblasts and osteoclasts) rapidly respond to circulating signals, altering global levels of bone formation and resorption respectively, and thus impacting bone homeostasis.^1–3^ The effects of adiponectin in bone have been researched in multiple conditions; however, these studies report variable outcomes with little explanation. Further exploration of adiponectin signalling is essential to fully understand the possibility of promoting or inhibiting its actions during ageing or disease. Of note, very few studies have examined the effect of adiponectin on osteoclasts, so we understand much less about its role in bone turnover. Here we explore the current literature on adiponectin in bone, looking in depth at the comparison between human and murine in vivo and in vitro data in health, with age and in disease, with reference (where possible) to the adiponectin isoform described.

## Adiponectin

**Fig. 1:**
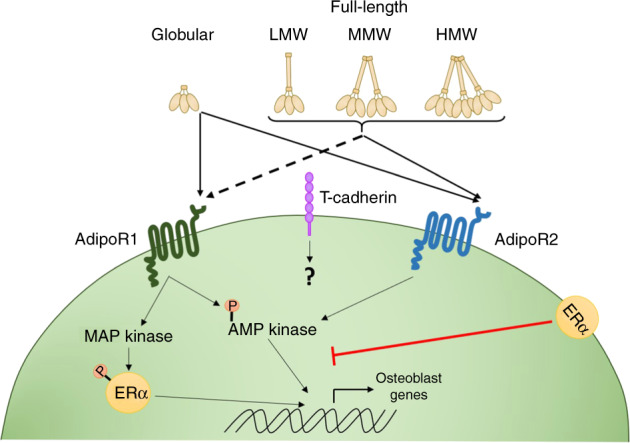
Adiponectin signalling. The full-length adiponectin protein can be cleaved into smaller active components, which circulate either as it’s globular domain^7^ or as full-length homo-complexes—low molecular weight (LMW) trimers, medium molecular weight (MMW) hexamers or high molecular weight (HMW) oligomers.^8–10^ Adiponectin can bind to two classical adiponectin receptors 1 and 2 (AdipoR1 and AdipoR2)^14,15^ leading to downstream signalling through several pathways, primarily driving AMP kinase (AMPK) and to a lesser extent activating MAP kinase (MAPK).^16–18^ Expression of the oestrogen receptor (ERα) appears to skew adiponectin receptor signalling to primarily trigger the MAPK pathway,^61^ which in turn phosphorylates both ERα and transcription factor SP1, altering downstream signalling. AdipoR1 has a higher affinity for the globular domain of adiponectin, whilst AdipoR2 displays an intermediate affinity for all adiponectin isoforms.^21^ In addition to the classical adiponectin receptors, HMW and MMW adiponectin can also interact with T-cadherin (cadherin-13; CDH13),^19,20^ although the downstream signalling and functional outcomes from these interactions are currently unknown

Adiponectin is the most abundant circulating adipocyte-secreted adipokine found in blood serum (5–15 µg/mL).^4,5^ The full-length protein (244 amino acids^6^) can be cleaved into smaller active components,^7^ which circulate either as it’s globular domain^7^ or as full-length homo-complexes referred to by their differing molecular weights (MW): a trimer (low MW; LMW); a hexamer (medium MW; MMW) or an oligomer (high MW; HMW) (Fig. 1).^8–10^ At a cellular level, adipocytes within the bone marrow adipose tissue (BMAT) and white adipose tissue (WAT) produce all of these adiponectin isoforms.^11^ For example, similar levels of adiponectin gene expression were observed in murine adipocytes isolated from WAT or BMAT.^11^ Yet protein expression was reportedly lower in rat total BMAT lysates^12^ and higher in adipocytes isolated from BMAT from rabbits and healthy 30-year old humans,^11^ compared to WAT. As such adipocytes from both tissues can contribute to the local levels of adiponectin within the bone (and bone marrow), as well as the circulating levels of adiponectin that are more commonly reported in studies. Indeed, BMAT levels positively correlate with total serum adiponectin levels in humans.^13^ Whilst, the relative contribution of each adipose tissue to the levels of adiponectin in the bone is unclear, and may change with age and disease (as discussed in the later sections), many studies speculate that due to its proximity BMAT acts as the largest contributor of adiponectin to the local bone levels.

Adiponectin mediates its effects through adiponectin receptors 1 and 2 (AdipoR1 and AdipoR2)^14,15^ leading to downstream signalling through several pathways including AMP kinase (AMPK), PI3K/protein kinase B, MAP kinase (MAPK), STAT3 (signalling transducer and activator of transcription 3) and ceramidase activation (Fig. 1).^16–18^ In addition to the classical adiponectin receptors, HMW and MMW adiponectin can also interact with T-cadherin (cadherin-13; CDH13).^19,20^ However, AdipoR1 and R2 knockout mice have a near-complete lack of adiponectin signalling,^21^ thus the functional relevance of T-cadherin in mediating the effects of adiponectin is currently unclear. Moreover, potential signalling mechanisms downstream of adiponectin–T-cadherin interactions have not yet been fully explored. Crucially, evidence suggests that the individual adiponectin receptors have differing binding efficacies for the different isoforms of adiponectin; with a murine myocyte cell line demonstrating higher affinity of AdipoR1 for globular adiponectin, whilst AdipoR2 displays an intermediate affinity for all adiponectin isoforms.^14^ Thus, it is likely that these differing protein–receptor interactions results in divergent cellular responses to adiponectin, even within the same cell. In addition, many studies explore serum levels rather than the tissue-level expression of adiponectin, and whilst the bone is well vascularised,^22^ understanding the link between serum levels and local tissue effects has proved challenging.

## Adiponectin receptor expression in cells of the bone

Within the bone, precursor bone cells, osteoblasts and osteoclasts have all been reported to express the adiponectin receptors, although conflicting findings exist that are important to consider when interpreting adiponectin-signalling responses from in vitro studies. For instance, AdipoR1 and R2 mRNA and protein are detectable in murine osteoblasts,^23^ human osteoblast precursors (bone marrow stromal cell- BMSC and osteoblast-like cell lines)^24,25^ and osteoclast precursors (human peripheral blood monocytes, PBMC).^26,27^ In all cases, adiponectin receptor 1 was detected at significantly higher levels (up to 100-fold) than adiponectin receptor 2. Indeed in some studies expression of AdipoR2 was below the limits of detection—e.g. no AdipoR2 protein was detected in healthy human tibial osteoblasts^28^ nor gene expression observed in the MC3T3 murine osteoblast precursor cell line.^29^ The absence or lower expression of adiponectin receptor 2 on bone cells suggests that they preferentially response to globular adiponectin, which has a higher affinity for adiponectin receptor 1 than the other forms of adiponectin.^14^ Moreover, the majority of in vitro osteoblast models display increased expression of AdipoR1, but not AdipoR2, following differentiation, including Saos-2^25^ and C3H10T1/2,^24^ a response not seen when MC3T3 were used.^23^ In contrast, MC3T3 cells up-regulated AdipoR2 expression and down-regulated AdipoR1 expression upon differentiation.^23^ This difference in the expression pattern by MC3T3 cells upon differentiation may help to explain some of the confounding results observed between studies exploring osteoblast response to adiponectin. By contrast, in vitro osteoclastogenesis does not appear to affect the expression of adiponectin receptors at either the mRNA or protein level.^27^

The expression of adiponectin receptors by the main precursors and mature bone cells demonstrates that all have the potential to interact with and respond to adiponectin during the different stages of bone homeostasis. However, key questions remain: What is the impact of adiponectin signalling on bone formation and turnover? How are these adiponectin-mediated effects influenced by bone damage, with age and by inflammatory diseases? Can a greater understanding of adiponectin regulation of bone homeostasis lead to novel strategies to repair injured and damaged bone?

## Impact of adiponectin on osteoblastogenesis and activity

Bone marrow adipocytes continuously release adipokines into the bone niche, bathing all cells including osteoblast and osteoclast progenitors in adiponectin.^30,31^ Indeed, the limited available data indicates that within the bone marrow niche adiponectin acts to promote osteoblastogenesis, whilst simultaneously inhibiting osteoclastogenesis (Table 1).^24,32–35^ Addition of full-length^24,35^ or globular^36^ adiponectin induces the expression of the osteogenic-related genes osteopontin^24,36^ and alkaline phosphatase^24,35,36^ in the murine mesenchymal progenitor cell line, C3H10T1/2,^24^ the pre-osteoblast MC3T3-E1 murine cell-line,^35^ and in human adipose-derived stem cells (ADSC) in vitro.^36^ Moreover, BMSCs from 5 week old adiponectin knockout mice exhibited reduced gene expression of key osteoblast promoting lysine specific histone demethylases (KDM4B and KDM6B) when compared to wildtype mice infused with either globular or full-length adiponectin.^37^ Absence of KDM4B and KDM6B reportedly increased gene expression of PPARG in human BMSC and thus switched the differentiation fate from osteogenic to adipogenic in vitro.^38^ Indeed, the presence of fatty bone marrow in adiponectin knockout mice was attributed to reduced KDM4B and KDM6B expression in BMSCs, triggering adipogenesis and ultimately causing a reduction in osteoblasts and increase in adipocytes on the trabecular surfaces.^37^ Furthermore, siRNA knockdown of AdipoR1 in C3H10T1/2 cells significantly reduced adiponectin-induced osteoblast differentiation in vitro.^24^ Supporting this, enhanced matrix mineralisation was observed in human ADSCs cultured in the presence of globular adiponectin compared to untreated controls.^36^ Of note, one study disagrees with the above literature: Kajimura et al., demonstrated that 6 week old adiponectin knockout mice have increased bone mass and osteoblast numbers, suggesting adiponectin inhibits bone mass accrual in young mice.^34^ However, no changes were observed in the gene expression of the osteoblast differentiation markers runx2 and osterix in 12-week-old bones from adiponectin knockout mice compared to the wildtype controls—suggesting that by this time point adiponectin was no longer able to limit bone formation.^34^ Importantly the mineralisation capacity of cells from these bones was not assessed. Bones from 10-day old adiponectin knockout mice exhibited increased cellular proliferation and reduced apoptosis.^34^ Indeed, treating serum-starved wildtype calvarial osteoblasts with either full-length or global adiponectin reduced proliferation and increased apoptosis rate over 24 h.^34^ Importantly, no changes in proliferation or apoptosis have been reported in the aforementioned studies, where various osteoblasts were cultured in the presence of serum.^24,35,36^ Serum starvation, therefore, may account for the discrepancies in proliferation and apoptosis observed between these studies and the Kajimura et al. Overall, adiponectin appears to have a predominantly positive role in boosting pre-osteoblast differentiation and mineralisation capacity, potentially protecting the bone by inducing formation and repair.

**Table 1:** In vitro effects of adiponectin on bone cells

Adiponectin Type	Species	Cell type	Effect	Ref
Full-length	Mouse	C3H10T1/2	↑ OPN mRNA ↑ ALP mRNA	^24^
	Mouse	MC3T3	↑ Mineralisation	^35^
	Mouse	RAW624	↓ Differentiation ↑ Apoptosis	^32^
	Human	Mononuclear osteoclast precursor	↓ Differentiation ↑ Apoptosis	^35^
	Mouse	CD14^+^ PBMC	↓ Resorption	^35^
Globular	Human	Adipogenic stem cells	↑ RUNX2, OPN, ALP mRNA ↑ Mineralisation	^36^
	Mouse	RAW264.7	↓ Resorption ↓ Osteoclast number ↑ Apoptosis	^39^
All	Mouse	Adiponectin KO BMSC	↓ Adipogenic differentiation	^37^
	Mouse	AdipoR1 siRNA KD C3H10T1/2	↓ Osteoblast differentiation	^24^

*AdipoR* adiponectin receptor 1, *ALP* alkaline phosphatase, *BMSC* bone marrow stem cells, *KD* knock down, *KO* knockout, *OPN* osteopontin, *PBMC* peripheral blood monocytic cells, *RUNX2* RUNX family transcription factor 2, *siRNA* short interfering RNA

## Impact of adiponectin on osteoclastogenesis and activity

By contrast, few groups have analysed the effect of adiponectin on osteoclastogenesis to date (Table 1). Addition of full-length adiponectin significantly inhibited the ability of human mononuclear cells and murine macrophage progenitors to differentiate into mature osteoclasts when cultured in vitro in the presence of osteoclastogenic stimulating factors [macrophage colony-stimulating factor and receptor activator of nuclear factor kappa-Β ligand (RANKL)].^39^ Similarly, globular adiponectin blocked RANKL-induced osteoclastogenesis of the murine monocyte cell line RAW264.7.^39^ Importantly these studies indicate that adiponectin reduces the ability of osteoclast precursors to mature. Akin to data on osteoblast precursors, global adiponectin treatment reduced the proliferation rate of RAW264.7 osteoclast precursors and increased their apoptosis through APPL1-mediated down-regulation of Akt1 activity.^39^ As such it is unclear whether adiponectin mediates a direct effect on osteoclastogenesis or an indirect effect by reducing overall precursor numbers. Additional studies are urgently required to reproduce these findings and clarify the supposed anti-osteoclastogenic role of adiponectin.

## Regulator of osteoblast and osteoclast progenitor migration

**Fig. 2:**
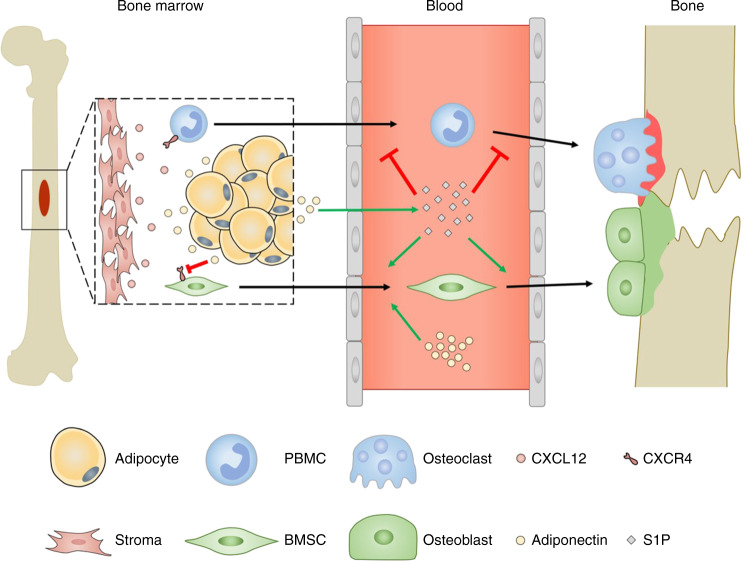
Adiponectin regulation of osteoblast and osteoclast migration. Osteoblast progenitors must exit the bone marrow niche to migrate towards the sites of resorption and/or damage. In fate mapping experiments, YFP expressing bone marrow-derived osteoblast precursors migrated to the endosteal surface to replenish osteoblast populations in healthy conditions and also in response to calvarial microfracture.^41^ Osteoblast and osteoclast progenitors express CXCR4 and migrate towards high levels of CXCL12 causing them to be retained in the bone marrow.^42^ Adiponectin directly^40^ and through increasing S1P in the serum,^17^ leads to increased osteoblast progenitor migration into the circulation and to bone in health,^48^ which is enhanced during injury.^41,116,117^ In contrast, S1P chemorepels osteoclast progenitors and osteoclasts,^51^ leading to decreased migration to damaged sites. This ultimately maintains the balance between resorption (red) and formation (green) to ensure structured bone repair

Osteoblast progenitors must exit the bone marrow niche to migrate towards the sites of resorption and/or damage (Fig. 2).^40^ Indeed fate mapping experiments have revealed that 70% of the osteoblasts found replenishing the endosteal surface during homeostasis originated from the bone marrow-derived osteoblast precursors.^41^ In addition, YFP-positive osteoblast precursors migrated from the neighbouring bone marrow to the site of calvarial microfracture, accounting for the majority of the cells at the site of injury after 7 days when compared to the bone resident pre- and mature osteoblast.^41^ This process can be controlled by the CXCR4-CXCL12 axis, where high levels of CXCL12 attract CXCR4 expressing cells and retain them.^42^ Globular adiponectin significantly reduces expression of CXCR4 mRNA in cultured murine BMSCs,^40^ therefore reducing the attraction of these cells to CXCL12 in the bone marrow. Similarly, adiponectin-deficient mice have higher numbers of CXCL12 positive cells within the bone marrow compared to wildtype mice,^40^ indicating that adiponectin regulates the exit of BMSCs from the bone marrow into the local bone environment where they can differentiate into osteoblasts.^40,43^ In addition, globular adiponectin enhanced BMSC migration across a Matrigel-coated Boyden chamber over 16h in vitro, which was coupled with an increase in MMP9 mRNA expression.^40^ Moreover, systemic globular adiponectin infusion increased serum CXCL12 levels and promoted nestin^+^ BMSC exit the bone marrow niche into the peripheral blood.^40^ Furthermore, therapeutic infusion of globular adiponectin significantly increased new bone formation at the site of calvarial injury, to a higher degree than seen in wildtype and adiponectin-deficient mice.^40^ This was attributed to enhanced migration of osteoblasts from the periphery to the injury site following adiponectin treatment, resulting in increased bone regeneration. Collectively these studies indicate that adiponectin regulates the CXCR4–CXCL12 axis within the bone marrow niche, facilitating the migratory exit of bone progenitor cells from this niche into the periphery in health and in response to injury. Comparably, osteoclast precursors such as PBMCs, express CXCR4^44^ and therefore osteoclast migration out the bone may mirror the mechanisms described for osteoblasts. Yet, few studies have examined the functional consequence of adiponectin on osteoclast migration, which are required to fully understand the overall impact of adiponectin on bone.

In addition to the CXCR4-CXCL12 axis, the bioactive lipid —sphingosine-1-phosphate (S1P)— has also been reported to influence the migration of osteoblast and osteoclast precursors (Fig. 2).^45,46^ S1P is synthesised intracellularly through the phosphorylation of sphingosine by the sphingosine kinases 1 and 2, and then trafficked out of the cell via sphingosine transporters where it interacts with S1P receptors to mediate its effects.^47^ Elevated levels of circulating S1P are observed in adiponectin-overexpressing transgenic mice.^17^ However, very little is known about the possible consequence of adiponectin on S1P-regulated migration. Both osteoblast and osteoclast precursors express S1P receptors (S1PR1, S1PR2).^48–50^ In addition, BMSC migrate towards S1P in vitro.^48^ However, osteoclast precursors chemorepel from S1P, either through S1PR1/2^50^ or S1PR2/3.^51^ In addition, sphingosine kinase 1 activity was upregulated in osteoclasts upon differentiation,^52^ indicating a role for osteoclast S1P release in maintaining the osteoclast-osteoblast balance. However, the impact of adiponectin in these responses remains unknown. One could postulate that direct effects of adiponectin signalling and adiponectin-induced increases in serum S1P cause the recruitment of the earliest bone precursors into the periphery, whilst simultaneously retaining pre-osteoblasts and mature osteoclasts within the bone to enable bone homeostasis and repair. Overall, these studies indicate that adiponectin increases osteoblast precursor migration, in part through CXCR4–CXCL12 axis and S1P, to favour bone formation. However, further work is required to fully understand these interactions and their functional consequences, as well as other potential molecular mechanisms underpinning adiponectin regulation of osteoblast and osteoclast migration.

## Role of adiponectin in the regulation of bone homeostasis

### In health and ageing

The actions of adiponectin in homeostatic bone turnover have been extensively explored in human and murine in vivo and in vitro studies in order to understand how it can be targeted in disease states (Tables 2 and 3). Of note, these studies overwhelmingly focus on juvenile or adult models, where BMAT and WAT are fully developed, and can both contribute to bone adiponectin levels. No studies to date have examined the role of adiponectin in foetal and neonatal bone development.

**Table 2:** In vivo effect of adiponectin in health, ageing and obesity

Condition	Species	Model	Adiponectin Type	Effect	Ref
Health	Mouse	homeostasis	AdipoR1 overexpression	↑ BMD ↓ TRAP5b	^33^
	Mouse	Adenovirus induced adiponectin overproduction	Full-length	↑ Bone mass, ↓ NTx	^35^
	Mouse	Bone explants	Adiponectin KO	↓ Trabecular/Cortical bone	^39^
	Mouse	homeostasis	AdipoR1 KO	↓ Trabecular bone ↓ Osteoblast number	^53^
Ageing	Human	67.2 ± 13.9^a^	–	Serum adiponectin ≠ Bone Strength	^65^
	Human	Post menopause	–	↑ Serum adiponectin ↓ BMD	^57^
	Mouse	OVX	Adiponectin KO	↓ Bone damage	^58^
	Rabbit	OVX with HA implant	Full-length	↓ Bone loss	^59^
	Human	40 ± 11.5^a^	–	↑ Serum adiponectin = ↓Bone Strength	^64^
	Mouse	5 months	Adiponectin-Tg overexpression	↑ Serum adiponectin = ↓Bone Strength	^66^
Obesity	Human	Weight loss	–	↑ Serum adiponectin ↓ β-CTX collagen	^71^

*AdipoR1* adiponectin receptor 1, *β-CTX collagen* collagen β c-terminal telopeptide; *BMD* bone mineral density, *HA* hydroxyapatite, *KO* knockout, *NTx* cross-linked N-telopeptides of type I collagen, *OVX* ovariectomy, *Tg* transgenic

^a^Years as mean ± SD

**Table 3:** In vivo effect of adiponectin in disease

Disease	Species	Model	Adiponectin Type	Cell	Effect	Ref
OA	Human	–	–	–	↑ Serum adiponectin in erosive OA	^86^
	Mouse	STR/Ort	–	–	↑ Serum adiponectin = ↓ Severity	^87,118^
Periodontitis	Mouse	Experimental periodontitis	Adiponectin KO	–	↑ Osteoclast number, ↓ Bone strength	^89^
			Globular	–	↑ Osteoclast number, ↓ Bone loss	^89^
	Mouse	In vitro LPS inflammation	Globular	RAW624	↓ TNF–α and IL1β ↑ IL10	^90^
RA	Human	–	Full-length	–	↑ Serum adiponectin	^91^
	Human	–	Full-length	–	↑ Plasma adiponectin	^91,92^
	Human	Ex vivo culture	Unknown	Osteoblast	↓ Osterix mRNA ↑ OPG mRNA	^94^
	Human	Ex vivo culture	Unknown	Osteoclast	↑ MMP9 & TRAP mRNA	^94^
	Mouse	CIA	Full-length	–	↑ Damage, ↓ BMD, ↑ Osteoclast number	^98^
	Mouse	CIA	Globular	–	↓ BMD	^99^
	Mouse	CIA	Unknown	–	↓ Score	^101^
	Mouse	CIA	Full-length	–	↓ Score, ↓ MMP3	^102^
Osteosarcoma	Human	16-year-old patient	–	–	↑ Adiponectin mRNA in diseased bone	^105^
	Mouse	In vitro	Globular	Saos-2	↑ Viability	^106^
	Mouse	In vitro	AdipoR inhibitor	Saos-2	↓ Proliferation	^108^
Cancer metastasis	Mouse	5TGMI cell cancer	Adiponectin KO	–	↑ Bone damage	^111^
			L-4F (HMW adiponectin)	–	↓ Bone damage	^111^
OI	Murine	*Col1a1* mutation	–	–	↓ Body fat	^113^
Osteopetrosis	Murine	Obesity induced	–	–	Exercise = ↓ Aberrant bone growth	^114^
	Human	Infantile osteopetrosis	Full-length	BMMC	↓ Adiponectin release ↓ Adipogenic differentiation	^115^

*AdipoR* adiponectin receptor, *BMMC* bone marrow mononuclear cells, *BMD* bone mineral density, *CIA* collagen induced arthritis, *HMW* high molecular weightb, *LPS* lipopolysaccharide, *MMP* matrix metalloproteinase, *KO* knockout, *OA* osteoarthritis, *OI* osteogenesis imperfecta, *OPG* osteoprotegerin, *RA* rheumatoid arthritis, *Score* clinical score, *SRT/Ort* murine strain prone to OA

Global overproduction of full-length adiponectin significantly increases trabecular bone mass and reduces bone resorption (as measured by plasma cross-linked N-telopeptides of type I collagen (nTx) levels) over a 2-week period in young 8 week old mice.^35^ Similarly, higher bone mineral density (BMD) and reduced number of TRAP5b-positive osteoclasts were observed in aged (56 weeks) AdipoR1-overexpressing transgenic mice, but not in younger mice aged 8 or 32 weeks.^33^ Likewise, AdipoR1-deficient mice have decreased trabecular bone volume, thickness, number and spacing, along with reduced osteoblast numbers.^53^ Similarly, 36 week old adiponectin knockout mice also display severe low bone mass affecting all skeletal elements, yet this was linked to decreased number and proliferation ability of osteoblasts rather than effects on osteoclasts.^34^ Interestingly osteoclast numbers were unaffected by loss of AdipoR1 in young mice aged 4 and 30 weeks.^53^ Collectively, these studies suggest that age may significantly alter the effects of adiponectin on osteoclasts. Moreover, they suggest that adiponectin-signalling through AdipoR1 is critical for maintaining osteoblast survival and activity with age and may also regulate osteoclast apoptosis.

**Fig. 3:**
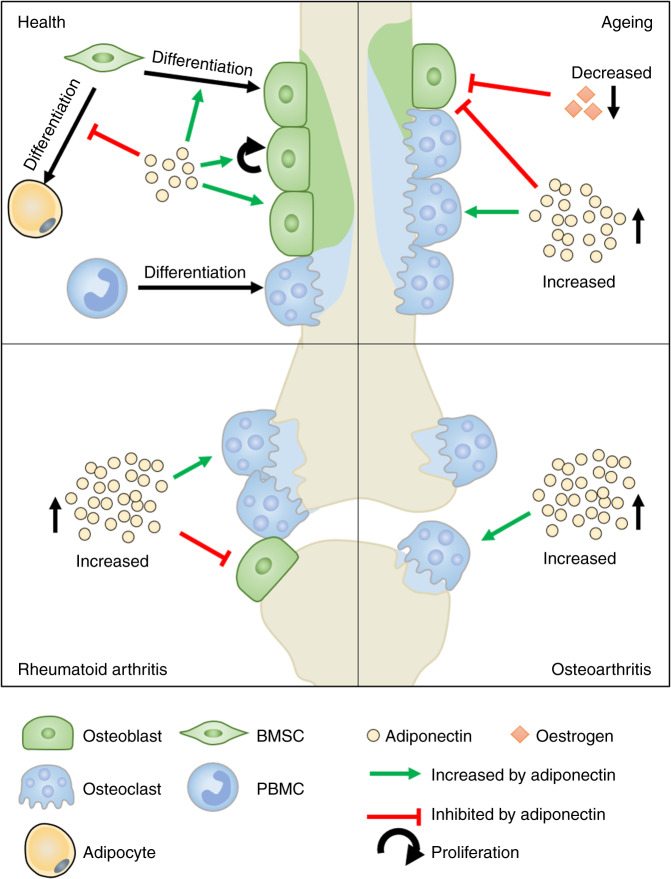
Adiponectin signalling in bone. Under healthy conditions, adiponectin supports the proliferation, migration, mineralisation and survival of osteoblasts (green), whilst decreasing osteoclastogenesis and resorption (blue), ultimately leading to bone homeostasis. By contrast, these traits are broadly dysregulated in bones as we age and in patients with obesity or diseases such as rheumatoid arthritis and osteoarthritis: Increases in adiponectin, or changes in environmental cues (including levels of oestrogen) that impact the downstream signalling pathways triggered by adiponectin (e.g. MAPK vs AMPK), pathologically tip the balance in favour of bone resorption and damage (blue) in these conditions

Mirroring the in vivo findings described above, bone explants from 3-day old wildtype mice implanted subcutaneously into the flank of adiponectin knockout mice had reduced trabecular volume and cortical bone parameters 2–4 weeks after implantation when compared to those implanted into wildtype mice,^39^ indicating that systemic levels of adiponectin were sufficient to impact normal bone remodelling. In vitro studies support these findings, with full-length adiponectin stimulating bone matrix deposition and hydroxyapatite formation by MC3T3-E1 cells, and reducing its resorption by CD14^+^ PBMC osteoclast precursors.^35^ Collectively these data indicate that adiponectin promotes bone formation and limits bone resorption in healthy young and old mice (Fig. 3A), where exogenous adiponectin may serve to maintain bone mass during age-related bone loss. Moreover, therapeutically adjusting the circulating levels of adiponectin may result in beneficial effects on the bone, avoiding the need to develop drugs that specifically target the bone. Importantly, no studies have yet explored the role of adiponectin in the development and maturation of healthy human bones either in vivo or ex vivo.

Both BMAT and serum adiponectin increase significantly with age^54^ and in osteoporosis^55,56^ and are broadly thought to negatively correlate with BMD in humans (Table 2). For example, post-menopausal women have higher serum adiponectin levels and reduced BMD compared to pre-menopausal women of the same age.^57^ Interestingly, a lack of adiponectin protects young mice from ovariectomy-induced bone loss 10 weeks after surgery.^58^ In contrast, sustained local release of low levels of therapeutic full-length adiponectin, from a hydroxyapatite-Matrigel implant, protected against ovariectomy-induced trabecular bone loss in the mandibles of rabbits.^59^ These studies suggest that the absence or consistently maintained low levels of adiponectin protect against menopause (hormonal) induced bone loss, maintaining BMD (Fig. 3B). Indeed, this would mitigate against the elevated levels of adiponectin anticipated in response to the increased BMAT levels observed with age (as reviewed by ^60^). This contrasts with healthy young bone (as discussed above), where higher levels of adiponectin are thought to promote bone formation and limit bone loss to maintain BMD. The exact mechanisms regulating the differential effects of adiponectin with age/in post-menopausal state remains unclear and requires further investigation. One possible explanation is the ability of the oestrogen receptor (ERα) to influence the downstream signalling response from the adiponectin receptors — where the presence of ERα triggers the MAPK pathway, rather than activating AMPK as would normally occur (Fig. 1).^61^ Given these changes occur in the absence of oestrogen^61^ as would be seen in post-menopausal women, it is unlikely that it is signalling through the ERα is responsible for these changes, but rather highlights the possibility that ERα acts as a co-receptor for the adiponectin receptors to alters the downstream signalling that is observed in response to adiponectin. Importantly, murine ovariectomy increased ERα mRNA expression in multiple tissues,^62^ and therefore is it likely that elevated ERα expression promotes adiponectin signalling through the MAPK pathway in these tissues and potentially contributes to menopause-induced bone loss.

Age-related decline in BMD is associated with increased susceptibility to microfractures in humans,^63^ however very few studies have directly examined the effect of adiponectin on bone strength. High circulating levels of adiponectin negatively correlate with bone strength and reduced maximal load in middle-aged overweight men (mean ± S.D.—age 40 ± 11.5 years, BMI 25 ± 6.2).^64^ By contrast, no link has been found between circulating adiponectin levels and the frequency of fracture in the men over the age of 70 with an average BMI ∼26 in this correlative study.^65^ These limited data appear to suggest that circulating levels of adiponectin influences bone strength and risk of fracture at least in overweight middle-aged men. However, more detailed longitudinal human studies, in which confounders such as BMI are carefully controlled for, are required to fully dissect the interaction of circulating adiponectin levels with BMD and fracture rates across different age groups.

Even fewer in vivo and in vitro studies exist that have directly assessed bone strength in response to adiponectin. Agreeing with the human data, adiponectin-overexpressing transgenic mice exhibited lower bone mass and lower strength at 2, 4^33^ and 5^66^ months compared to age matched wild-type controls. In addition treating 3-D cultures of human osteoblasts and osteoclast precursors in mineral forming spheres (known as osteospheres) with an unknown adiponectin isoform significantly reduced their strength, as assessed using nano-indentation; indicating weaker, more breakable bone.^67^ Mechanistic studies are required to elucidate the how the ageing process alters adiponectin signalling within the bone, but also systemically, to modulate BMD and strength.

### In obesity and obesity-related diseases

Circulating adiponectin levels are decreased in obesity in both adults and children (young and adolescent).^68,69^ This is in-part due to adipocytes in the WAT acquiring a pro-inflammatory phenotype, which alters their production of various adipokines including adiponectin.^70^ Whether adipocytes in BMAT acquire the same obesity-linked phenotype remains unclear. Interestingly, weight loss in obese adolescents was associated with increased serum adiponectin levels coupled to lower levels of the bone resorption marker, collagen β c-terminal telopeptide.^71^ Moreover, caloric restriction in mice^11,72^ or humans with anorexia nervosa^73–76^ has also been associated with an increase in bone marrow adiposity, coupled with increased adiponectin and a decrease in bone mass. Despite the link between obesity and reduced BMD (Table 2),^69,77,78^ most studies overlook adiponectin measurements in their analyses. Therefore, further research is needed to explore the impact of obesity in humans on adiponectin signalling in bone.

Whilst murine models can provide a more in-depth study of the effects of obesity on the bone, again none of the available literature explores the importance of adiponectin in the changes they describe. For instance, 12-weeks of high fat diet significantly reduced trabecular bone volume and number,^79^ and decreased the amount of cancellous bone, as well as collagen and osteoid expression observed after 23-weeks of the diet^80^ in wildtype mice — where these changes were linked to increases in adipocyte volume, and thus BMAT, rather than reduction in osteoblast or osteoclast numbers. Moreover, obesity significantly limited fracture repair, with no callus or connected bone observed in obese mice days after fracture unlike control animals.^81^ High levels of limb fractures are also observed in obese children,^82–84^ where abnormal BMAT and adiponectin levels may be contributing to reduced bone strengthen and delayed repair.

Obesity increases the risk of patients developing osteoarthritis (OA) and therefore, it is possible that changes in adiponectin may have detrimental effects on the bones of patients with OA (Table 3).^77,78,85^ Human studies have revealed higher levels of adiponectin in a meta-analysis of patients with more advanced OA compared to BMI matched patients with new onset OA;^78^ and in the serum of women with erosive OA in the hand compared to non-erosive disease.^86^ In contrast, serum adiponectin concentrations negatively correlated with cartilage lesions and OA severity in adolescent (26 weeks old) STR/Ort mice, suggesting adiponectin has a protective role in a human-like murine OA model.^87^ It is difficult to make any definitive conclusions based on the currently available data on whether changes in adiponectin levels represent part of the molecular mechanism driving pathology of OA, or are part of the protective response to the on-going joint damage. As such these concepts need to be explored further taking into account the aforementioned confounding factors.

### In inflammatory disease

#### Periodontitis

Periodontitis is the most common inflammatory bone disease worldwide, affecting 60% of >75-year olds.^88^ Despite this, we have a limited understanding of the importance of adiponectin in periodontal bone damage; with most of the evidence to date generated using murine models (Table 3). For instance, following induction of experimental periodontitis by coating molars with a bacterial broth, adiponectin knockout mice have increased osteoclast numbers in palatal bone compared to wildtype control mice.^89^ Moreover, globular adiponectin therapy reduced the number of TRAP^+^ osteoclasts in the inflamed palatal bone in adiponectin knockout mice, leading to a concomitant reduction in alveolar bone loss.^89^ In vitro, RAW264 osteoclast precursors treated with lipopolysaccharide (LPS) and globular adiponectin showed reduced pro-inflammatory cytokine secretion (TNF-α and IL-1β) and increased anti-inflammatory IL-10 release, compared to LPS treatment alone.^90^ Clearly further studies are required, but the data available suggest that adiponectin exerts a direct anti-osteoclastogenic role during periodontitis.

#### Rheumatoid arthritis

Patients with rheumatoid arthritis (RA) have elevated levels of adiponectin in plasma^91,92^ and synovial fluid,^93^ compared to sex, age and BMI-matched control samples. These findings correlate with radiographic erosions suggesting a role for adiponectin in disease pathology (Table 3).^92,93^ Moreover, adiponectin stimulated MMP9 and TRAP expression in cultured human osteoclasts from RA patients^94^—unlike what has been reported for healthy osteoblasts (see above). Importantly these data suggest that the chronic inflammatory environment in RA switches the adiponectin response in osteoclasts from being anti-osteoclastogenic to a pathological pro-resorptive response. However, osteoblasts isolated from trabecular bone of RA patients cultured with adiponectin showed reduced mRNA expression of osteoblast differentiation marker osterix and increased osteoprotegerin (RANKL competitive inhibitor).^94^ Overall these data indicate that adiponectin contributes to bone damage in rheumatoid arthritis: where it directly reduces osteoblast differentiation, whilst stimulating osteoclastogenesis leading to enhanced bone resorption (Fig. 3B). It is unclear what specific factors linked to the chronic conditions of rheumatoid arthritis are responsible for the reversal of the effects of adiponectin on osteoblasts and osteoclasts. It is highly likely to be a consequence of the multi-factorial pathological changes that occur within the joint, such as reduced adiposity within the synovium and BMAT,^95^ the chronic production of pro-osteoclastogenic cytokines (TNFα and RANKL^96^) and pro-inflammatory cytokines (e.g. IL-1β) that influence expression of AdipoR1 in the synovium,^97^ which act in concert to either mask the anti-osteoclastogenic effects of adiponectin or to alter adiponectin receptor-mediated signalling in an as yet unknown manner.

Despite this, preclinical animal studies have been inconclusive (Table 3). Therapeutic treatment with full length^98^ or globular adiponectin^99^ exacerbated juxta-articular bone erosions in collagen induced arthritis models, with a concomitant reduction in BMD,^98,99^ bone volume to trabecular volume ratio,^98^ and trabecular number^98^ seen when compared to untreated mice^99^ or to mice treated with osteopontin short hairpin RNA.^98^ These pathological effects of adiponectin therapy have been attributed to an increase in osteoclast number and osteoclast activity — as measured by enhanced levels of RANKL mRNA expression.^99^ Moreover, therapeutic administration of functional blocking antibodies against MMW (KH7-33), or both MMW and HMW (KH4-8) isoforms of adiponectin, after disease onset significantly decreased arthritic clinical score and degradation in the joint cavities, when compared to arthritic mice treated with PBS.^100^ Whilst no specific bone parameters were measured, KH4-8 treatment also led to a reduction in serum RANKL concentrations when compared to controls,^100^ suggesting reduced osteoclast numbers and activity, and potentially bone damage in these animals. By contrast, therapeutic treatment of arthritic mice with an unknown isoform of adiponectin^101^ or full-length adiponectin^102^ at first signs of inflammation reduced the histopathological score (a composite measure of inflammatory cell infiltration, synovial hyperplasia, and bone destruction) when compared to untreated controls. Of note, these studies did not directly measure changes in bone anatomy following adiponectin therapy, therefore it is unclear whether the reduction in histopathological score observed equated to reduced bone damage. Supporting this possibility, the collagen-matrix degrading metalloproteinase, MMP3, was expressed at significantly lower levels in arthritic joints treated with adiponectin than the contralateral PBS treated joints.^102^ There are clear experimental differences between the studies cited above, which might account for the discrepancies in the reported effects. For example, the studies reporting pro-arthritic effects administered adiponectin a maximum of 3 times a week, whereas mice displaying anti-arthritic responses were given daily injections of adiponectin. Such different treatment regimens will alter the absolute concentration of adiponectin experienced by cells at a given time and thus their responses, which in turn will impact the pathological responses observed. Further research is urgently needed to clarify the role of adiponectin therapy on bone damage in murine models of inflammatory arthritis.

## Cancer and rare bone diseases

### Cancer

Tumour growth within the skeleton can occur through multiple mechanisms, including the inappropriate expansion and activity of osteoblasts (osteosarcoma)^103^ or more commonly as a result of tumour metastasis, often seen in advanced breast cancers.^104^ Assessing the impact of adiponectin in osteosarcoma is very challenging: primary tumours are extremely rare (1 in 100,000) and osteosarcoma cell lines (e.g. Saos-2 and MG-63) are frequently used as “normal” immortalised osteoblasts in in vitro studies.^105^ As such limited data exists on adiponectin effects on bone cancer development and progression (Table 3). RNA-sequencing analysis revealed higher adiponectin gene expression in the bone from a single patient with primary osteosarcoma compared to the patient’s own healthy bone tissue,^105^ although analysis of more patient samples is required to make any definitive conclusions. In vitro, globular adiponectin increased the viability of the human Saos2 osteosarcoma cell line, but reduced the activity of the pro-mineralisation enzyme, alkaline phosphatase, in supernatants.^105^ By contrast, the adiponectin receptor agonist, AdipoRon, decreased the proliferation of Saos-2 cells in vitro.^106^ Given that AdipoR1 has a higher affinity for globular adiponectin, it is possible that increased signalling through AdipoR1 compared to AdipoR2 may promote abnormal osteoblast proliferation contributing to osteosarcoma, whilst equal signalling through both receptors may be important to control normal proliferative responses in healthy bone. Indeed, the loss of AdipoR1 has been linked to reduced osteoblast survival in vitro,^24^ and absolute number in vivo^53^ under healthy conditions (as described — *In health and ageing*) and may represent a novel therapeutic strategy to treat osteosarcoma. It is important to note that no data are currently available on the bone phenotype in AdipoR2 deficient mice or cells. These concepts require further investigation.

Tumour metastasis to the bone results in bone damage, increased pain and is a leading cause of death in breast cancer patients.^61,103,107^ Divergent adiponectin responses have been reported for tumour lines in vitro based on their expression of the oestrogen receptor (ER). The non-invasive ER^+^ MCF-7 breast cancer cell line demonstrated increased proliferation response to globular adiponectin.^61^ By contrast, treatment with full-length^108,109^ or globular^61^ adiponectin reduced cell proliferation,^61,108,109^ and increased apoptosis^109^ of the invasive ER^−^ MDA-MB-231 breast cancer cell line. Of note, adiponectin-induced signalling through the AMPK pathway in ER^−^ MDA-MB-231 cells, whilst MAPK pathway was triggered in ER^+^ MCF-7 cells.^61^ However, the effects of adiponectin on the migratory capacity of ER- cells and their ability to metastasise to the bone remains unclear; with studies reporting an increased migration of ER- cells using globular adiponectin, but no effect upon full-length adiponectin treatment.^110^ Moreover, inoculation of normal mice with established multiple myeloma (5TGM1) cells intravenously induced bone damage in wildtype mice, which was further exacerbated in adiponectin knockout mice.^111^ Furthermore, increasing HMW adiponectin levels by administering an apolipoprotein memetic peptide, L-4F, dramatically reduced bone lesions and damage, and concomitantly increased trabecular bone volume in myeloma-bearing mice compared to vehicle treated controls.^111^ Collectively, these data indicate that adiponectin can protect against multiple-myeloma-induced bone damage, potentially by acting directly on the tumour cell (5TGM1 cell) to induce apoptotsis.^111^ Clearly additional research is required to dissect the importance of oestrogen signalling and adiponectin-mediated tumour migration, as well as the contribution of different adiponectin isoforms to these responses and whether particular isoforms may be therapeutically beneficial to patients with osteosarcoma.

### Osteopetrosis and osteogenesis imperfecta

Patients with rare bone diseases, such as osteogenesis imperfecta (OI) and osteopetrosis, have unorganised bone structure and increased bone frailty,^112^ which can severely reduce the quality of life of those affected. The role of adiponectin in these diseases has been sparsely researched (Table 3) and requires further investigation. In patients with OI, mutations in COL1A1 or COL1A2 result in increased bone fragility.^113^ Moreover, *Col1a1* mutant mouse have reduced overall body fat content,^113^ suggesting that these mice have lower levels of adiponectin — although this was not explicitly measured. Similarly, an osteopetrosis-like phenotype is induced in mice maintained on the obesity-inducing high carbohydrate diet,^114^ and where adiponectin concentrations are presumably much lower (as discussed above in the obesity section). Conversely, exercise-induced weight loss significantly reduced aberrant bone growth in osteopetrotic mice and re-established bone homeostasis,^114^ indicating that increasing adiponectin levels may have therapeutic benefit in osteopetrosis. Additionally, BMSC from infantile osteopetrosis patients were unable to differentiate into adipocytes, secreting lower levels of adiponectin compared to BMSC isolated from healthy age-matched donors.^115^ Neither study actually measured adiponectin, yet it seems highly probable that fluctuations in its concentrations may contribute to changes in the bone. A greater understanding of adiponectin signalling and regulation of normal bone structure and strength in these rare diseases is urgently needed-based on our current knowledge treatment with adiponectin may alleviate some of the symptoms that patient’s experience.

## Conclusion

Under healthy conditions, adiponectin supports the proliferation, migration, mineralisation and survival of osteoblasts, whilst concomitantly limiting proliferation, migration and survival in osteoclasts. On balance, this allows adiponectin to promote bone formation and limit bone resorption. By contrast, these traits are broadly dysregulated in bones following menopause and in patients with obesity or diseases such as chronic inflammation and cancer, where the loss of adiponectin, or changes in environmental cues that impact the downstream signalling pathways triggered by adiponectin (e.g. MAPK vs AMPK) pathologically tip the balance in favour of bone resorption and damage (Fig. 3). However, inconsistencies in study design and outcome measures, along with discrepancies in the observations made in animals and humans make it difficult to draw definitive conclusions that can be utilised clinically. Indeed, in-depth, large-scale studies exploring adiponectin levels in multiple bone diseases, accounting for confounding factors such as age and BMI, are needed to fully understand how adiponectin interacts with bone. Furthermore, more detailed mechanistic studies are required to understand the interaction of the different adiponectin isoforms with each of the receptors, and the impact which oestrogen receptors and other unknown molecules have on downstream signalling and the functional consequences of these. These studies are critical to further our understanding of the beneficial and pathological roles of adiponectin for a specific context, and the efficacy of targeting adiponectin or its signalling therapeutically to treat bone abnormalities and induce repair.
